# Comparison of the Outcomes Between Bilateral Thoracoscopic Maximal Thymectomy Versus Trans-Sternal Maximal Thymectomy for Non-Thyomomatous Myasthenia Gravis Patients: A Local University Hospital Experience

**DOI:** 10.7759/cureus.12288

**Published:** 2020-12-25

**Authors:** Waseem M Hajjar, Naief W Almasry, Abdulaziz S Alarifi, Fahad B Alfahad, Khalid A Alanazi, Abdullah f Alfaleh

**Affiliations:** 1 College of Medicine, King Saud University, Riyadh, SAU

**Keywords:** autoimmune disease, complete stable remission, myasthenia gravis, stable pharmacological remission, surgical approaches

## Abstract

Introduction: Myasthenia gravis (MG) is an autoimmune disease characterized by excessive and intense weakness of both respiratory and skeletal muscles. Management of MG involves both medical and surgical treatment. The surgical management includes resection of the thymus gland by many approaches, either bilateral thoracoscopic maximal thymectomy (BTT) or trans-sternal maximal thymectomy (TS). We hypothesized that bilateral thoracoscopic maximal thymectomy is as effective as trans-sternal maximal thymectomy to treat and control the disease.

Objective: This study aimed to compare the two approaches (BTT and TS) and determine which is better in terms of outcomes.

Methodology: A retrospective cohort study was conducted among 50 myasthenia gravies patients; 30 patients underwent bilateral thoracoscopic maximal thymectomy (BTT) and 20 were operated by trans-sternal maximal thymectomy (TS). The study was conducted at King Khalid University Hospital (KKUH), Riyadh, Saudi Arabia, between 2007 and 2017.

Result: The mean age of the MG patients was 32.6 years, ranging from 14 to 75. Thirty-four (68%) patients were females, and 16 (32%) were males. The BTT showed less operation time (P<0.0001) and less intubation time (anesthesia time), which was statistically significant (P<0.0001). Hospital stay and ICU stay were both reported to be less in BTT (4.03 and 0.37, respectively) with p-values of 0.006 and 0.0001, respectively.

There was no significant association between all categorical study variables and the MG patients’ outcome (BTT/TS) in terms of mortality, morbidity, complete stable remission, pharmacological remission, and complications.

Conclusion: Bilateral thoracoscopic maximal thymectomy is as effective as trans-sternal maximal thymectomy to control and treat the disease.

## Introduction

Myasthenia gravis (MG) is a chronic autoimmune neuromuscular disease characterized by excessive and intense weakness in the respiratory and skeletal muscles. There are two ways to manage MG: medical and surgical [1]. Medical management includes the use of medications like pyridostigmine (cholinesterase inhibitor), corticosteroids, and azathioprine, but it does not usually show any significant improvement without other types of intervention. On the other hand, surgical management is considered the first essential and effective line in treating the thymomatous or non-thymomatous MG [2-4]. Currently, surgical management can be performed by many different surgical approaches, either by bilateral thoracoscopic maximal thymectomy (BTT) or by trans-sternal maximal thymectomy (TS) [2]. However, both approaches are effective in treating patients with MG [1-2,4]. The purpose of a maximal thymectomy operation is to remove completely the thymus gland, in addition to excise the ectopic thymic tissue, which is embedded in the mediastinal fat lying in the neck and the anterior mediastinum [5-6].

We hypothesized in this study that bilateral thoracoscopic thymectomy (BTT) for non-thymomatous MG patients is as effective as trans-sternal maximal thymectomy to treat and control the disease. We also hypothesized that bilateral thoracoscopic thymectomy has less complications, less morbidity and mortality, but has more cost-effective and cosmetic results.

## Materials and methods

A retrospective cohort study was carried out at King Khalid University Hospital (KKUH), Riyadh, Saudi Arabia, between 2007 and 2017 among the MG patients who underwent BTT or TS. Fifty-seven patients were evaluated; but 50 adult patients with non-thymomatous MG were included, while seven patients with thymoma or anterior mediastinal mass were excluded. All preoperative and postoperative use of medications was recorded. The comparison between the two approaches (BTT and TS) included several parameters: operation time, blood loss, hospital stay, ICU time, intubation time (anesthesia time), complications (pneumonia, wound infection etc.), diaphragmatic paresis or paralysis and vocal cord paralysis. Patients’ postoperative follow up was monitored through regular visits to the outpatient’s clinic post-operative for more than 24 months. Complete (cure) stable remission was also included in the comparison, which is defined as patients having no obvious MG signs or symptoms and not on any medications after six months from the surgery. Pharmacological stable remission means that the patients’ signs and symptoms have been controlled after the surgery, but the patient is still on some medications. The study approval was obtained from the institutional review board (IRB), Research Ethics Committee, College of Medicine, King Saud University (KSU). The confidentiality and privacy of the patients' medical records were secured and assured.

Surgical procedures

BTT is performed while the patient is under general anesthesia, at a supine position, and intubated by a double-lumen tube with single lung ventilation. The approach is usually from the right axillary side with three ports, 5, 5, and 10mm. The thymus gland is dissected and excised completely, through the video-assisted thoracoscopy technique. On the left side, one 5 mm port is used to visualize the left phrenic nerve during dissection of the left thymic lobe. 

TS is done under general anesthesia and at a supine position. A single lumen endotracheal tube is used for the patients' intubation and ventilation, a midline median sternotomy incision carried out dividing the sternum, and the two components of the thymus gland, the neck and the chest, are usually completely excised with the anterior mediastinal fat from the lower neck level down to the diaphragm. At the end of the procedure, the sternum is closed using sternal wires after draining the pleural cavity by two chest tubes.

## Results

A total of 50 patients’ medical records were reviewed; 34 (68%) patients were females, and 16 (32%) were males, with a mean age (SD) of 32.6 (12.2) years, mean body weight 74.97 (13.61) kg, and mean height 164.5 (7.69) cm. Thirty patients (60%) underwent BTT and 20 (40%) underwent TS. All patients in both groups underwent a maximal thymectomy by removing completely the thymus gland in its two components, the neck and the chest with the anterior mediastinal fatty tissue. Approximately 48% of the patients were on complete stable remission, and 52% were on stable pharmacological remission, with a mean hospital stay (SD) 5.18 (3.72) days, and a mean amount of blood loss (SD) 127.30 (74.6) ml. None of the patients from both groups had major complications, nor needed ventilator support. Two patients (4%), one from each group, developed transient unilateral diaphragmatic paresis.

The operations took in average mean duration time (SD) 2:04 (0:78) hours, and only one patient (2%) needed a blood transfusion. Almost all patients needed preoperative steroids (94%), after that they were referred to ICU with a mean (SD) ICU time of 0.84 (0.933) days and intubation time mean (SD) of 2:84 (0:88) hours, and they were followed for a mean (SD) of 24.44 (20.51) months, as shown in Table 1.

**Table 1:** Socio demographic and clinical characteristics of myasthenia gravies patients. (n=50). (kg) kilogram, (cm) centimeter, (ml) milliliter, (ICU) intensive care unit,(sd) standard deviation, (N) number of cases, (TS) Trans-sternal maximal thymectomy, (BTT) Bilateral thoracoscopic maximal thymectomy.

Variables		
Age (years)	Mean(SD)	32.6 (12.2)
Body weight (kg)		74.97(13.61)
Height (cm)		164.5(7.69)
Hospital stay (days)		5.18(3.72)
Amount of blood loss (ml)		127.30(74.6)
Operation time (hours)		2:04(0:78)
ICU stay (days)		0.84(0.933)
Intubation time (hours)		2:84(0:88)
Follow-up period (months)		24.44(20.51)
Gender (male)	N (%)	16(32.0)
Amount of removed thymo-fatty tissue (All)		50(100)
Complete stable remission		24(48.0)
Pharmacological stable remission		26(52.0)
Complications (pneumonia, wound infection)		0(0)
Ventilator support		0(0)
Diaphragm paresis (transient)		2(4.0)
Vocal cord paralysis		0(0)
Mortality		0(0)
Morbidity		0(0)
Blood transfusion		1(2.0)
Preoperative Steroid use		47(94.0)
Type of surgery ( BTT)		30(60.0)
Type of surgery (TS)		20(40.0)

The t-test was performed to examine the difference between patients who underwent TS or BTT operations. The results were statistically significant in both operation time and intubation time, where BTT operation took much less time than TS (t (49) = 4.570, P<0.0001), and much less intubation time (t (49) = 4.690, P<0.0001). Hospital stay and ICU stay were both reported to be less in BTT 4.03, 0.37 with p-values of 0.006 and 0.0001, respectively. The results were insignificant for all other tested variables (age, body weight, height, amount of blood loss, and follow up period), as shown in Table 2.

**Table 2:** Comparison of Mean values of quantitative study variables in relation to type of surgery of myasthenia gravies. (TS) Trans-sternal maximal thymectomy, (BTT) Bilateral thoracoscopic maximal thymectomy, (kg) kilogram, (cm) centimeter, (ICU) intensive care unit, (ml) milliliter.

Variables	Type of surgery		Mean difference	t-value	P-value	95% Confidence interval of the mean difference	
	TS	BTT				lower	upper
Age (years)	32.7(13.9)	32.5(11.17)	0.200	0.056	0.955	-6.955	7.35
Body weight (kg)	74.86(12.5)	75.06(14.51)	-0.202	-0.051	0.960	-8.18	7.78
Height (cm)	164.3(6.5)	164.7(8.49)	-0.325	-0.145	0.885	-4.83	4.18
Operation time (hours)	2:56(0:69)	1:69(0:64)	0.871	4.570	0.0001	0:48	1:25
Hospital stay (days)	6.9(2.1)	4.03(4.14)	2.866	2.855	0.006	0.847	4.885
ICU stay (days)	1.55(0.94)	0.37(0.56)	1.18	5.050	0.0001	0.703	1.663
Amount of blood loss (ml)	140(82.97)	118.8(68.7)	21.166	0.982	0.331	-22.16	64.49
Intubation time (hours)	3:44(0:80)	2:45(0.69)	0.991	4.690	0.0001	0:566	1:41
Follow up period (months)	28.38(22.74)	22.61(19.55)	5.777	0.836	0.408	-8.196	19.75

In both approaches, there were no statistically significant association between MG patients’ outcome and all categorical study variables, such as; gender, amount of removed thymo-fatty tissue, complete stable remission, pharmacological stable remission, and most complications (pneumonia, wound infection), diaphragm paralysis or paresis, vocal cord paralysis, ventilator support, blood transfusion, preoperative steroid use, mortality, and morbidity), as shown in Table 3.

**Table 3:** Association of categorical study variables with the type of surgery of myasthenia gravis. (TS) Trans-sternal maximal thymectomy, (BTT) Bilateral thoracoscopic maximal thymectomy, (N) number of cases.

Variables		Type of surgery				Χ^2^ value	P-value
		TS		BTT			
		N	%	N	%		
Gender	Male	6	37.5%	10	62.5%	0.061	0.804
	Female	14	41.2%	20	58.8%		
Complete stable remission		9	37.5%	15	62.5%	0.300	0.765
Pharmacological stable remission		8	30.8%	18	69.2%	2.739	0.098
Complications (pneumonia, wound infection)		0	0%	0	0%	-	-
Ventilator support		0	0%	0	0%	-	-
Diaphragm paresis	Yes	1	50%	1	50%	0.087	0.768
	No	19	39.6%	29	60.4%		
Vocal cord paralysis		0	0%	0	0%	-	-
Blood transfusion	Yes	1	100%	0	0%	1.531	0.216
	No	19	38.8%	30	61.2%		
Preoperative steroid use	Yes	18	38.3%	29	61.7%	0.946	0.331
	No	2	66.7%	1	33.3%		
Mortality		0	0%	0	0%	-	-
Morbidity		0	0%	0	0%	-	-

## Discussion

Surgical management is considered one of the important keystones and essential or effective line in treating thymomatous or non-thymomatous MG patients [1].

However, there are two surgical approaches, bilateral thoracoscopic maximal thymectomy (BTT) and trans-sternal maximal thymectomy (TS). Few previous studies in the literature have compared both surgical approaches regarding the post-operative outcomes and the intra-operative parameters and reported different findings. Diaz and his colleagues mentioned that the video-assisted thoracoscopic surgery (VATS) approach achieved more complete stable remission than the TS maximal thymectomy approach [7]. Gung and his colleagues reported that the VATS approach has more operative time and low blood loss than the TS approach [8]. Jurado and Shiono [9,10] agreed with Gung and his colleagues [8] regarding the operative time, but on the contrary, they reported that the TS approach has more blood loss compared with the thoracoscopic approach. Zahid et al. [3] demonstrated different results than Gung and his colleagues [8] and mentioned that VATS approach has shorter operative time compared to the TS approach. Additionally, most of the results of several studies of [1,8,9] showed that VATS has less pharmacological remission and hospital stay compared to TS. Regarding the ICU stay, it was reported in most of the studies that VATS has less ICU stay [4,9]. Moreover, the incidence of complications (e.g., pneumonia) is lower in VATS and there is no significant difference in wound infection between VATS and TS [8]. Mayer et al. [2] reported that the patients who underwent TS needed more post-operative ventilation, but it was not statistically significant. Additionally, the overall mortality is higher in patients who underwent TS surgery with no statistical significance [9]. Regarding the morbidity, mortality, and post-operative complications, most of the studies found there is no difference observed between the two groups [1,4,8,10,11].

The present study found that BTT has less operative time and less intubation time (anesthesia time) than TS. In addition, it has a favorable cosmetic factor. However, there was no statistical difference in complete stable remission, pharmacological remission, blood loss, and complication between the two approaches. Our study results coincide with most of the other studies, which found that the VATS group had shorter operative time [3,8,10], but disagreed with other studies that found no time difference between the two groups [2,6,9,11]. 

In addition, our study results showed significant statistical difference between the two approaches regarding hospital stay, which agreed with others [12-15], who reported that VATS has a shorter hospital stay. Also, the present study results show a significant statistical difference between BTT and TS in terms of ICU stay, which was found to be less in BTT and which correlated with other study results, where they found that VATS has a shorter ICU stay than TS [4,9].

The current results of our study found that there is no difference between BTT and TS concerning complete stable remission, while the results of two other studies [7,16] indicated that VATS has more complete stable remission compared to TS. Our results also reported no statistical difference between the two groups regarding pharmacological remission, which disagrees with Bachmann et al. [1], who mentioned that VATS has less pharmacological remission than TS. Additionally, in the present study, the two groups didn’t show any difference regarding blood loss but the results of previous studies [12,17] reported that VATS has less blood loss compared with TS. The present study results showed no difference between the two groups regarding mortality and morbidity, which agreed with other studies [11,18] that reported no difference in mortality and morbidity and agreed with several studies [1,4,8,10] that found no difference between the two approaches in morbidity. Other studies [19,20] reported that VATS reduced mortality. 

## Conclusions

BTT is as effective as TS maximal thymectomy to treat and control the disease, but it is more cost-effective than TS in terms of hospital stay, ICU stay, and also has a favorable factor on cosmetic factors. BTT has less operative time and intubation time (anesthesia time) than TS, and the results were statically significant.

There is no significant difference between the two approaches regarding complete stable remission, stable pharmacological remission, blood loss, mortality and morbidity, and all other tested variables.
